# Molecular Insights of New Psychoactive Substances (NPS) 2.0

**DOI:** 10.3390/ijms242417492

**Published:** 2023-12-14

**Authors:** Annagiulia Di Trana, Angelo Montana, Alfredo Fabrizio Lo Faro, Francesco Paolo Busardò, Simona Pichini

**Affiliations:** 1 National Centre on Addiction and Doping, National Institute of Health, 00161 Rome, Italy; annagiulia.ditrana@iss.it; 2 Department of Biomedical Science and Public Health, University Politecnica delle Marche, 60121 Ancona, Italy; angelomontana49@gmail.com (A.M.); fabriziolofaro09@gmail.com (A.F.L.F.);

The New Psychoactive Substances (NPS) phenomenon represents an ever-changing global issue, with a number of new molecules entering the illicit market every year in response to international banning laws. While it has been confirmed that the most-represented class per analogue number and for consumers is synthetic cannabinoid receptor agonists (SCRAs), newly emerging compounds active on opioid receptors have recently been reported, representing an unknown challenge for health specialists and toxicologists [1].

Indeed, as a consequence of the recent international ban on fentanyl analogues and their synthetic precursors, the so-called nitazenes, benzimidazole opioids were proposed as a potent alternative to the more popular fentalogues causing several fatal and non-fatal intoxications [2,3]. Another new class of NPS that emerged as an alternative to SCRAs has sparked the attention of the scientific community and international agencies: semi-synthetic cannabinoids. These molecules, such as hexahydrocannabinol (HHC) or delta-8-tetrahydrocannabinol (Δ8-THC), are synthetic derivatives of the naturally occurring active principle of *Cannabis sativa*, delta-9-tetrahydrocannabinol (Δ9-THC) and cannabidiol (CBD). Although the pharmacological potency of semi-synthetic cannabinoids appears to be lower than that of SCRAs, their misuse potential is very high due to the ease with which they can be manufactured and their legal status. However, little is known about their pharmacological profiles, representing a new challenge for forensic and clinical toxicologists [4,5].

In this complex scenario, improving molecular knowledge in the field of NPS is crucial to elucidate their toxicological properties and set up effective preventive strategies for facing this international public health problem. In particular, this Special Issue focuses on some of the most recent updates on NPS toxicity, in vitro pharmacological studies on NPS, and the development and validation of new analytical methods for detecting NPS in biological matrices and their application to real cases. 

De Simone and colleagues aimed to investigate the neurotoxicity of MAM-2201, a naphthoyl-indole derivative belonging to the SCRA class. To this end, the authors developed a novel 3D cell culture model to obtain a model that mimicked the complexity of the physiological and biochemical properties of brain tissue better than the classic 2D models. Specifically, human astrocyte spheroids, generated from the D384 astrocyte cell line, were treated with different MAM-2201 concentrations (1–30 µM) and exposure times (24–48 h). Interestingly, the study demonstrated that MAM-2201 induced cytotoxicity to astrocyte spheroids, showing that the investigated molecule affected cell growth and viability, size and morphological structure, E-cadherin and extracellular matrix, CB1-receptors, glial fibrillary acidic protein, and caspase-3/7 activity in a concentration- and time-dependent manner.

New insights into NPS neurotoxicity were also studied by Daziani et al., who reviewed the most recent literature on synthetic cathinones’ neurotoxicity. The authors collected evidence on synthetic cathinones’ involvement in different neurological events, including increased alertness, mild agitation, severe psychosis, hyperthermia, and death. According to the 76 studies included in the systematic review, synthetic cathinones’ neurotoxicity seems to exert brain-related adverse effects, including encephalopathy, coma and convulsions, and sympathomimetic and hallucinogenic toxidromes, together with the risk of developing excited/agitated delirium syndrome and serotonin syndrome.

Nunez-Montero and colleagues validated a new gas chromatography–tandem mass spectrometry (GC-MS/MS) method for quantifying 4-chloromethcathinone (4-CMC), N-ethyl pentedrone (NEP), and N-ethyl hexedrone (NEH, also named HexEN) in oral fluid (OF) and sweat in order to assess the pharmacokinetics of six consumers following the oral administration of 100 mg of 4-CMC and the intranasal administration of 30 mg of NEP and NEH. The samples were liquid/liquid-extracted, dried under nitrogen flow, derivatized with pentafluoropropionic acid, and finally resuspended in 50 μL of ethyl acetate. The method was fully validated according to international guidelines. The study showed that NEP and NEH were absorbed within the first hour, while 4-CMC reached its maximum concentration peak in the first three hours. Concerning the sweat samples, the total excreted amount of 4-CMC and NEP reached about 0.3% of the administered dose, whereas the total amount of NEH excreted in sweat 4 h after administration was approximately 0.2% of the administered dose.

A synthetic cathinone’s disposition in sweat was also the focus of Di Giorgi et al.’s investigation, aiming to determine the excretion of methylone and its metabolites in sweat following the ingestion of increasing controlled methylone doses. To this end, the authors conducted a clinical trial involving twelve healthy volunteers, who were administered 50, 100, 150, and 200 mg of methylone. Methylone and its metabolites 4-hydroxy-3-methoxy-N-methylcathinone (HMMC) and 3,4-methylenedioxycathinone (MDC) were analyzed in sweat patches via liquid chromatography–tandem mass spectrometry (HPLC-MS/MS). Methylone and MDC were detected in sweat at 2 h and reached their highest accumulation (C_max_) at 24 h after the administration of 50, 100, 150, and 200 mg doses. In contrast, HMMC was not detectable at any time interval after each dose. Sweat proved to be a suitable matrix for methylone and its metabolites’ determination in clinical and toxicological studies, providing a concentration that reveals recent drug consumption.

Specifically concerning methylone, the plasma kinetics of this synthetic cathinone were studied by Poyatos and colleagues, describing, for the first time, methylone’s and HMMC’s plasma pharmacokinetics following the controlled administration of 50–200 mg of methylone to 12 male volunteers. In this regard, the authors set up a randomized, crossover, double-blinded, placebo-controlled study, with a total of 468 plasma samples collected. Before the trial, a new LC-MS/MS method was validated to quantify methylone and its metabolites in plasma. The samples were liquid/liquid-extracted, and the supernatant was fortified with 0.1 mL of acidified methanol and evaporated under nitrogen. The samples were reconstituted with a mobile phase and injected into the LC-MS/MS instrument. The methylone plasma concentrations increased in a dose-proportional manner, as demonstrated by the increasing maximum concentration (Cmax) and area under the curve of the concentrations (AUC). Methylone exhibited rapid kinetics, with a T_max_ of 1.5 h for the 50 mg dose and approximately 2 h after all the other doses. HMMC exhibited faster kinetics compared to methylone, with lower bioavailability than the parent compound. Unexpectedly, methylone’s pharmacokinetics were linear for 50–200 mg oral doses in humans.

It must be acknowledged that NPS consumption is not always intentional due to the unexpected addition of these substances to common drugs of abuse. Indeed, drug adulteration with NPS is a very common practice carried out by drug manufacturers that may occur at different stages of drug production. Adulterants found in different drugs change over time in response to different factors. To this end, Di Trana et al. performed a systematic literature search to review recent studies on drug adulteration with NPS, with toxicological evidence both in biological and non-biological matrices. Although the obtained sample analyses revealed the presence of well-established adulterants such as levamisole for cocaine or paracetamol/acetaminophen for heroin, the reported data disclosed new adulteration practices, such as the use of NPS as cutting agents for classic drugs of abuse and other NPS. For example, heroin adulterated with synthetic cannabinoids or cocaine adulterated with fentanyl/fentalogues raised particular concerns. Notably, adulterants play a role in some adverse effects commonly associated with the primary drug, such as levamisole-adulterated cocaine, which may induce vasculitis via an autoimmune process. Hence, it is essential to constantly monitor adulterants due to their changing availability, which may threaten drug consumers’ health.

In conclusion, this Special Issue provides updated studies and reviews concerning the most recent aspects of NPS issues, their toxicity, and pharmacokinetic profiles, aiming to contribute to the general knowledge in the field in order to highlight the importance of the continuous investigation of this still-unclear field by the scientific community and health professionals.
